# From Metrics to Meaning in Neurological Rehabilitation: Clinicians’ Perspectives on Digital Metrics of Upper Limb Functioning—A Focus Group Study

**DOI:** 10.2196/87339

**Published:** 2026-06-24

**Authors:** Johannes Pohl, Laura Mayrhuber, Olivier Lambercy, Chris Easthope Awai

**Affiliations:** 1 Data Analytics and Rehabilitation Technology (DART), Lake Lucerne Institute, Rubistrasse 9, Vitznau, 6354, Switzerland, 41 0774868083; 2 Rehabilitation Engineering Laboratory (RELab), Department of Health Sciences and Technology, ETH Zurich, Zürich, Switzerland

**Keywords:** digital metrics, upper limb, kinematic metrics, real-world performance metric, clinical reasoning, neurorehabilitation

## Abstract

**Background:**

Digital assessment technologies, such as optical motion capture and inertial measurement units, enable detailed kinematic analysis and continuous monitoring of upper limb activity in persons with neurological conditions. While such *digital metrics of functioning* are increasingly recognized in research, their uptake in clinical neurorehabilitation is limited. It remains unclear which *digital metrics of functioning* clinicians perceive as most meaningful and how these are integrated into patient-centered care. Understanding clinicians’ information needs and reasoning processes is a prerequisite for implementing digital assessment technology.

**Objective:**

This study aims to characterize how rehabilitation professionals perceive, prioritize, and integrate *digital metrics of functioning* into clinical reasoning and to identify features that would support their routine use.

**Methods:**

Three 90-minute focus groups were conducted in 3 Swiss neurorehabilitation centers, involving 11 clinicians with diverse professional backgrounds (5 physiotherapists, 4 occupational therapists, 1 movement scientist, and 1 medical practitioner). Participants discussed essential parameter domains and individually rated the relevance and meaningfulness of 17 kinematic metrics for the well-studied drinking task and 10 established arm use performance metrics. Verbatim transcripts were analyzed using reflexive thematic analysis, and rating data were summarized descriptively.

**Results:**

Five main themes were identified. (1) *Functional requirements to interpret movement quality and performance* (active/passive range of motion, strength, selective muscle control, and grasp) form the basis for interpreting movement. (2) *Essential aspects of movement quality* (smoothness, efficiency, and compensatory movement) are valued when aligned with observable task execution. (3) *Added value of real-world performance* (hourly activity profiles, arm-use symmetry, and functional workspace) represents the reference for patient-centered reasoning. (4) *Individualizing what matters*, including diagnosis-specific preferences, shapes assessment selection. (5) *Blending clinical eye and reference data* reflects clinicians’ reliance on visual judgment complemented by normative values. Intuitive metrics such as task duration, number of movement units, and range of motion were favored, whereas confidence was lower in more complex metrics (eg, jerk and interjoint coordination).

**Conclusions:**

Clinicians value intuitive *digital metrics of functioning* when they are clearly linked to patient-centered outcomes and supported by normative references. The findings highlight the need for targeted educational strategies and digital competency training that help clinicians interpret digital metrics and integrate them with contextual information and clinical reasoning.

## Introduction

Upper limb dysfunction is among the most disabling consequences of neurological conditions such as stroke and spinal cord injury (SCI). Loss of dexterity and limited arm use reduce independence in activities of daily living (ADL) and restrict participation in social and professional roles [1,2]. The International Classification of Functioning, Disability and Health (ICF) captures these interactions under the umbrella term “functioning,” which encompasses body functions, activities, and participation [3]. Assessment of upper limb functioning is therefore central to motor rehabilitation, informing both patient-centered goal setting [4] and comprehensive recovery monitoring [5,6].

Guidelines for clinical assessments after stroke emphasize the routine application of established core sets to ensure that clinically relevant and meaningful domains of functioning are systematically evaluated [7-9]. In conventional clinical assessments, clinicians evaluate the patient’s maximal voluntary capacity, relying on observation and standardized rating scales to assess constructs such as active range of motion (ROM) and patient-reported ADL performance. These methods are often integrated into the decision-making process known as clinical reasoning, which clinicians use to interpret information and guide treatment. However, these assessments are limited in their ability to adequately and reliably capture movement quality and detect subtle changes [10,11]. Additionally, upper limb capacity assessments often fail to align with the actual performance in real-world settings. Multiple studies show a capacity-performance mismatch [12,13] and indicate that gains on standardized tests do not always translate into increased arm use [14,15].

To address these limitations, technology-based assessments have been developed to reliably quantify upper limb movements with high temporal resolution. In analogy to the ICF framework, we introduce a technology-based counterpart: *digital metrics of functioning*. These metrics are derived from technologies such as optical motion capture, computer vision, and wearable sensors, which enable the quantification of fine-grained movement execution and real-world activity. *Digital metrics of functioning* can be grouped into 2 complementary domains. In our framework, movement quality is the overarching construct that characterizes how a movement is executed. It can be evaluated through observational movement analysis (clinician-rated) or instrumented kinematic analysis, the latter yielding kinematic metrics that quantify aspects of movement quality such as smoothness, movement time, and compensatory movements during both standardized and habitual tasks. In standardized tasks, these metrics reflect what an individual can perform under controlled conditions, that is, upper limb capacity.

In contrast, *real-world performance metrics* reflect arm use behavior in daily life, for example, through average acceleration or arm use symmetry, thereby capturing arm use in people with neurological conditions in a habitual environment.

Although related, *kinematic* and *real-world performance metrics* represent distinct constructs. Improvements in task-based kinematic metrics, that is, movement quality or upper limb capacity, do not necessarily translate into arm use behavior in a habitual environment. Moreover, real-world performance may be influenced by contextual, motivational, and behavioral factors beyond the assessed movement quality and capacity under controlled conditions [15,16]. By integrating these different domains into clinical practice, digital measures could provide information beyond observer-based ratings [17,18] and enable clinicians to identify meaningful patterns, such as a potential mismatch between increased capacity and reduced real-world arm use performance [15,19]. For instance, by capturing both *digital metrics of functioning* (ie, *kinematic* and *real-world performance metrics*) and clinical assessments, individuals requiring special attention can be identified, including those who show a mismatch between increased functional capacity and declining real-world performance. These people may benefit from personalized rehabilitation that includes monitoring arm use performance, feedback, and targeted interventions [20]. This underscores the value of multidomain assessments in detecting such discrepancies and guiding intervention planning.

Among *kinematic metrics*, those quantifying the movement quality of reach-to-grasp movements have been validated most extensively [21]. The reach-to-grasp function is critical for independence in daily life [22,23] and provides insights into recovery mechanisms [11,16]. The drinking task, a standardized and meaningful ADL, has been recommended by international expert consensus for assessing upper limb movement quality in both research and clinical contexts [11]. A set of validated *kinematic metrics* exists for individuals with stroke or SCI, as well as healthy populations [24-26].

*Real-world performance metrics* are particularly valuable for monitoring arm use behavior in uncontrolled, ecologically valid environments [27]. Established *real-world performance metrics* include measures of arm use duration and intensity [28,29], which are widely used in research but rarely in clinical settings [30]. Novel metrics, such as real-world reach-to-grasp quantification [31] or mapping functional reaching space [32], provide task-specific insights into the real-world performance of the upper limbs.

Both kinematic and real-world performance metrics are modulated by contextual and task-related constraints, such as health status, environmental factors, and personal factors, which influence movement execution [33,34] and everyday arm use [35] beyond underlying motor capacity. When operating in an ICF-based framework, clinicians need complementary information on health-related goals, environments, and individual behavior to integrate *digital metrics of functioning* into clinical reasoning.

Despite these advances, it remains insufficiently understood how established *digital metrics of functioning* are interpreted and integrated into everyday clinical reasoning by frontline clinicians. While international expert consensus initiatives have defined key metrics and assessment paradigms [8,9,11], less is known about how practicing rehabilitation professionals make sense of these metrics in relation to their clinical expertise and decision-making processes. To support user-centered adoption of digital assessment technologies, evidence is needed on how established *digital metrics of functioning* can be harmonized with clinicians’ empirical knowledge and contextual judgment. The objective of this study was to explore rehabilitation professionals’ perspectives on the use of *digital metrics of functioning* when assessing the upper limb in neurological conditions. In this context, relevant *digital metrics of functioning* are understood as those that convey actionable meaning for clinical understanding, decision-making, and communication in practice. By examining how clinicians interpret and integrate such metrics into clinical reasoning, this perspective complements existing consensus and validation work by adding the dimension of clinician-perceived interpretability and clinical utility. Specifically, we addressed the following research questions:

1. Which measurement constructs do clinicians consider essential when interpreting *digital metrics of functioning* of the upper limb (ie, *kinematic* and *real-world performance metrics*) in neurological rehabilitation?
2. Which specific *kinematic* and *real-world performance metrics* are regarded as relevant and intuitive for daily clinical practice?
3. Which complementary information is deemed important to facilitate the integration of *kinematic* and *real-world performance metrics* into clinical reasoning?

## Methods

### Study Design

We conducted a focus group study using reflexive thematic analysis (realist orientation) to explore opinions on metrics for functioning in the Swiss neurorehabilitation landscape. Focus groups were held between December 2023 and July 2024. All participants received verbal and written information about the study and provided written informed consent prior to participation. Participants also consented to the audio and video recording of the focus group discussions for research purposes. Data were anonymized during transcription, and any potentially identifiable details were removed before analysis and reporting.

### Ethical Considerations

According to the Swiss Federal Act on Research involving Human Beings (HRA), the study did not require formal ethical approval because it involved health care professionals only and did not collect health-related personal data. A formal determination of competence (Req-2025-009-30) was obtained after data collection, serving as administrative confirmation that the project was outside the scope of the HRA.

### Participants and Recruitment

Invitations were distributed via email, along with a recruitment flyer addressed to therapy leads and medical directors at 8 neurorehabilitation clinics. Clinics were asked to nominate at least 3 clinicians who treated neurological patients with upper limb impairments and had at least 3 years of clinical experience. Prior experience with technology-based assessment was described as beneficial but not required. Nonparticipation was mainly due to organizational constraints (ie, allocating staff for a 90-minute time commitment). Out of 8 invited clinics, 3 agreed to participate in a focus group discussion (one per site).

### Procedures

Focus groups, each lasting 90 minutes, were held in quiet meeting rooms at the participating clinics and guided by a moderator (JP, a physiotherapist with 10 years of experience in mixed clinical and research settings), who had no prior supervisory relationship with the participants. A co-moderator (LM, a physiotherapist) managed audio recordings, field notes, and whiteboard visualizations. Focus groups consisted of 3 to 4 participants. Smaller focus groups (3‐5 participants) are considered appropriate in health research when participants share specific expertise, as they allow more time per participant and facilitate open, more in-depth discussion [36,37].

**Figure 1.**
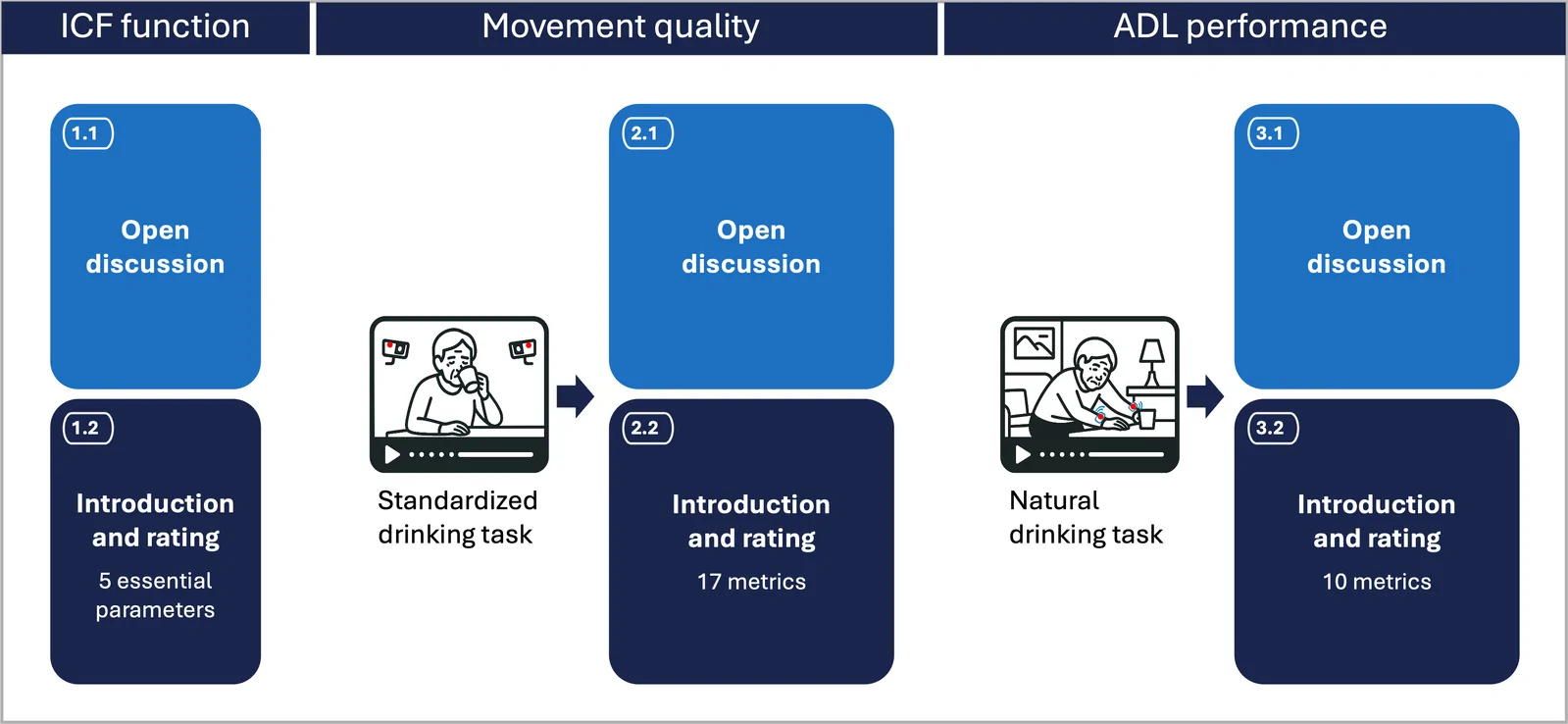
Structure of focus group discussions, including joined components of discussions before ratings. ADL: activities of daily living; ICF: International Classification of Functioning, Disability and Health.

Participants were familiarized with the principles of focus group discussions and the ICF as the conceptual framework for health assessments. The focus group discussions were conducted according to an interview guideline (Multimedia Appendix 1), which included key questions and practical patient examples, and were structured into 3 parts (Figure 1). Each part began with a discussion of parameters that are relevant and meaningful for quantifying the respective ICF domain, followed by participants indicating the importance of each parameter on individual handouts. Throughout this paper, the term “rating” denotes an indication of importance (yes/no selection) for each parameter.

The first part of the session focused on identifying key metrics within the ICF body function domain (32 items, including ROM, muscle strength, and somatosensory function; Table S1 in Multimedia Appendix 2). This section served as a warm-up to familiarize participants with the domain-specific reasoning process. To stimulate discussion on prioritization and underlying reasoning at the beginning, participants were limited to selecting a maximum of 5 metrics they considered most important in their clinical routines. As this exercise was intended to stimulate reasoning rather than generate interpretable quantitative rankings, the individual votes were not analyzed as outcomes. For transparency, the full table of items is provided in Table S1 in Multimedia Appendix 2.

The second part, focusing on kinematic metrics that quantify aspects of movement quality, began with a video clip showing a patient with moderate one-sided paresis performing the standardized drinking task from both frontal and sagittal perspectives. The drinking task was designed as a representative reach-to-grasp activity to provide a shared, standardized reference for discussing movement quality constructs; however, participants were encouraged to reflect on upper limb movement more broadly, beyond this specific task. Participants then discussed important movement characteristics, clinically relevant features, and requirements for clinical reasoning. The concept of “relevance” was intentionally not predefined or operationalized using explicit criteria. Instead, participants were invited to discuss *digital metrics of functioning* within clinical scenarios, allowing perceptions of relevance to be implied through how metrics were contextualized, interpreted, and integrated into clinical reasoning. In practice, participants appeared to associate relevance with aspects such as clinical interpretability, usefulness for decision-making, familiarity, feasibility in clinical workflows, and perceived patient-centered value.

Thereafter, participants indicated the perceived importance of a list of kinematic metrics for the drinking task [24,25], which are commonly categorized into the following domains: *velocity and movement time*, *movement smoothness and coordination*, *movement strategy*, and *ROM* (Table S2 in Multimedia Appendix 2).

The third part included key discussion points on upper limb performance during ADLs, prompted by video clips of the same patient performing the drinking task in a daily-life context. Metric importance was assessed across 10 performance metrics in the domains of *arm use duration*, *arm use intensity*, and *reaching count and space*. Metrics quantifying the duration and intensity of arm use have shown good measurement properties [29] and explain real-world performance well [28] (Table S3 in Multimedia Appendix 2). Despite the limited evidence base for validity, the metrics for reaching count and movement space were included based on previous reports of clinical professionals’ preferences [38].

### Data Analysis

The participants’ opinions were analyzed using a mixed methods analysis. In qualitative evaluations, reflexive thematic analysis was applied to transcriptions, while ratings were evaluated quantitatively by calculating, for each metric, the proportion of participants who selected it. In the ICF-function section, quantitative evaluations of ratings were excluded due to differences in rating logic.

The reflexive thematic analysis framework [39] enables an interpretative, flexible, and researcher-reflexive engagement with participants’ perspectives, making it particularly well suited to capturing how clinical experts construct clinical reasoning, decision-making, and value-based evaluation of upper limb functioning. Transcripts were imported into the text annotation software Taguette (v1.4.1) [40] and coded by 2 coders (JP and LM) from a realist perspective, assuming that the participants’ words represented their actual experiences and opinions. Codes were generated by adopting an inductive-deductive approach, combining data-driven coding with concepts from existing frameworks (Table 1). Both coders discussed the essential aspects, interpretations, and underlying theories of code labels in regular meetings. Codes were then structured into clusters, forming subthemes and superordinate themes, by 2 researchers (JP and LM), who collated coherent clusters. The meaning, reflexivity, and coherence of themes were then discussed between both researchers. Differences in coding or theme boundaries were addressed through deeper analysis of the coded data. The researchers explored how different interpretations reflected different aspects of participants’ opinions. Where divergent interpretations arose, both perspectives were discussed and revised as the analysis progressed.

Finally, verbatim German quotations and their English translations, together with contextual descriptions of the respective subthemes, were sent to participants for review. All participants confirmed that the translations accurately reflected the original meaning and context. This study was conducted and reported in accordance with the Standards for Reporting Qualitative Research (SRQR) [41]. The completed SRQR checklist, indicating where each item is addressed in the paper, is provided in Checklist 1.

**Table 1.** Coding process.

Step	Action
1. Inductive coding	Independent coding (JP and LM) capturing semantic meaning line-by-line. No fixed codebook or a priori agreement threshold was imposed. Discussion, merging, or retaining code labels whose semantics were judged “functionally identical.” Differences in coding were resolved with deeper analysis of the code and discussions of divergent interpretations until consensus was reached.
2. Deductive mapping	Code mapping of agreed labels to ICF^a^ and existing measurement taxonomies.
3. Theme construction	Code-clustering into preliminary themes (JP and LM) upon agreement for coherence, divergences, and reflexive commentary.
4. Integration of quantitative priorities	Alignment of priority ratings with qualitative themes in a joint display, where illustrative quotes explain *why* specific metrics were prioritized or deprioritized.

a ICF: International Classification of Functioning, Disability and Health.

## Results

### Study Sample and Focus Group Characteristics

Out of the 8 invited clinics, 3 participated in the focus group study, including a total of 11 participants across 3 focus groups (one conducted in each clinic). Participants presented a range of professional backgrounds, including physiotherapy, occupational therapy, movement science, and medical practice, and varied in clinical roles and experience levels (Table 2). Clinical experience ranged from 4 to 26 years, and research experience ranged from 0 to 20 years. Average durations of focus group discussions were 33 (SD 9.6) minutes for ICF functions, 25 (SD 1.5) minutes for kinematic metrics, and 15 (SD 3.5) minutes for real-world performance, respectively.

**Table 2.** Participant characteristics.

ID	Field	Specialization	Profession	Clinical experience in years	Research experience in years
P1-C1	Stroke, ND^a^, SCI^b^	Therapy sciences	PT^c^	6	20
P2-C1	Stroke, ND, SCI	Neurology	MP^d^	15	15
P3-C1	Stroke, ND, SCI	Neurorehabilitation	PT	4	1
P4-C2	SCI	Technology-assisted therapy	MS^e^	6	4
P5-C2	SCI	Upper extremity–focused therapy	MS	8	11
P6-C2	SCI	Upper extremity–focused therapy	PT	8	1
P7-C2	SCI	Upper extremity–focused therapy	OT^f^	18	0
P8-C3	Stroke, ND, SCI	CIMT^g^, PNF^h^	OT	26	3
P9-C3	Stroke, ND, SCI	Technology-assisted therapy	OT	3	0
P10-C3	Stroke, ND, SCI	Neurorehabilitation	PT	17	0
P11-C3	Stroke, ND, SCI	Neurorehabilitation	OT	4	0

a ND: neurodegenerative diseases.

b SCI: spinal cord injury.

c PT: physiotherapist.

d MP: medical practitioner.

e MS: movement scientist.

f OT: occupational therapist.

g CIMT: constraint-induced movement therapy.

h PNF: proprioceptive neuromuscular facilitation.

**Figure 2.**
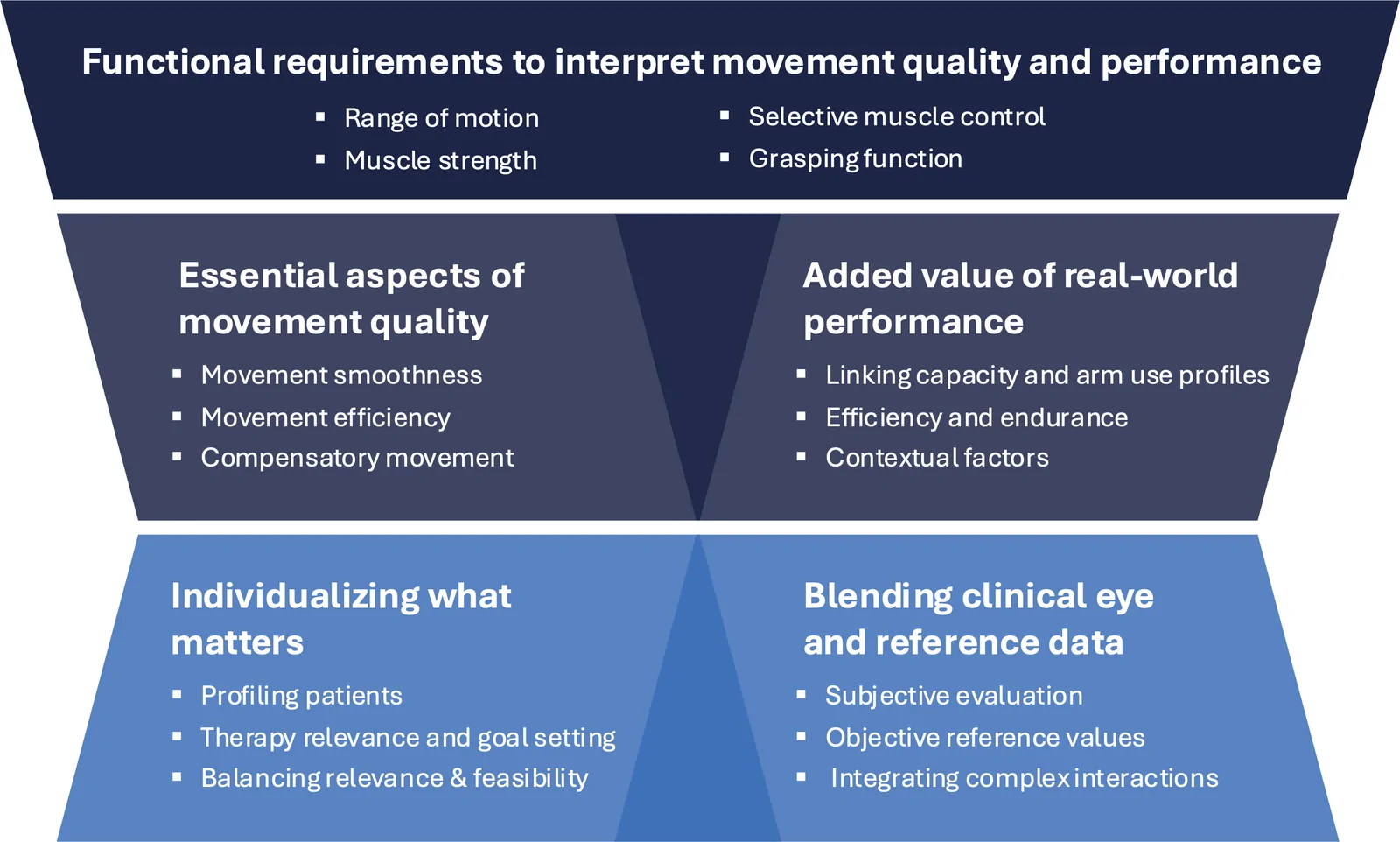
Themes and subthemes from the clinician focus group analysis illustrating the key dimensions guiding the interpretation and integration of *digital metrics of functioning*.

**Figure 3.**
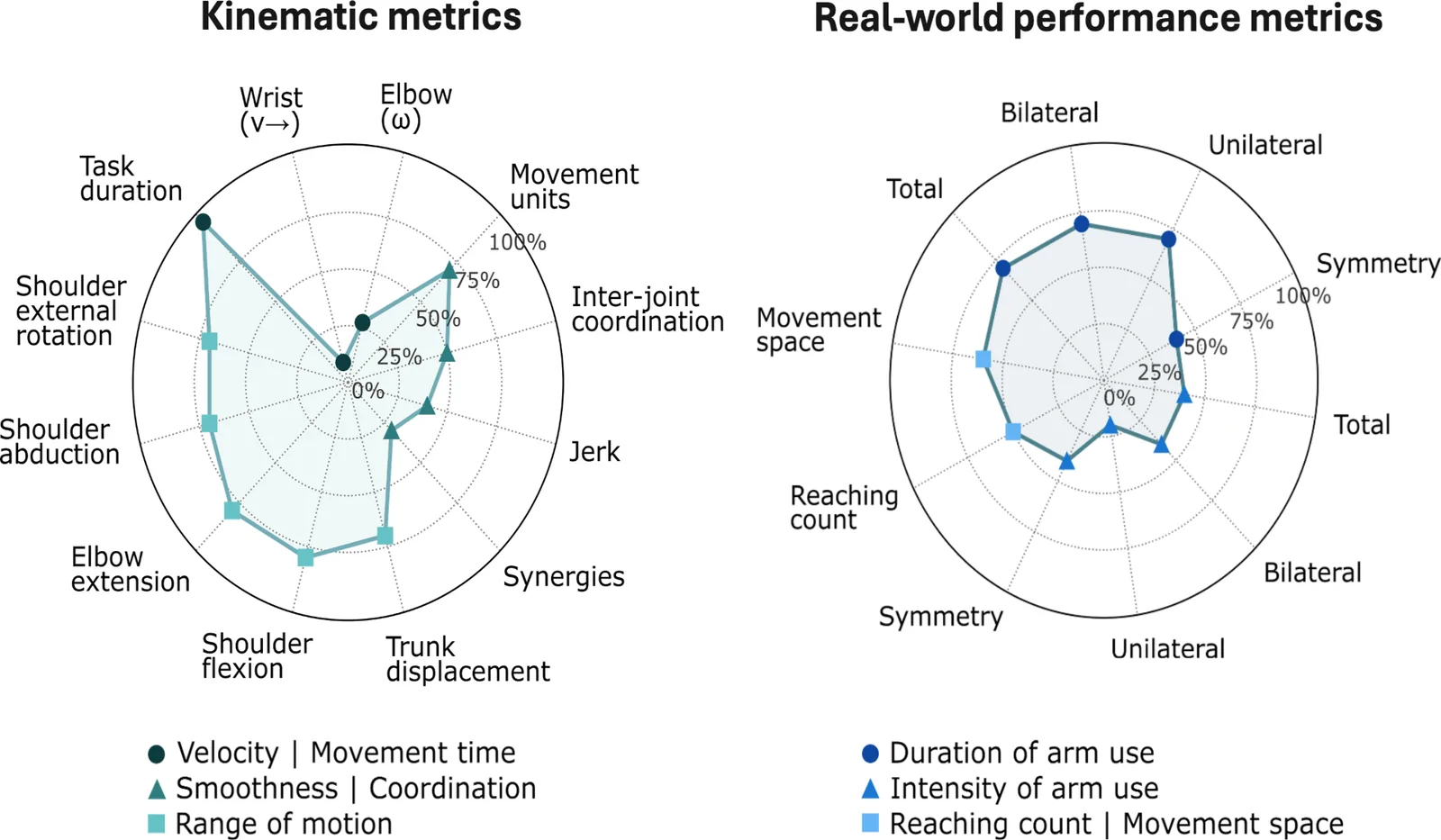
Radar plots representing the proportion of participants who rated each metric as meaningful and relevant for clinical practice. Kinematic metrics and real-world performance metrics, color-coded by metric domains. In the real-world performance panel, metrics with identical names (eg, symmetry) are distinguished by their domain (intensity vs duration), as indicated in the legend. Percentages represent the proportion of participants who rated each metric (0% at the center indicates no rating; 100% at the outer ring indicates all participants rated). The movement strategy domain was excluded from the *kinematic metrics* because no participant identified it as important. *v*→: velocity; *ω*: angular velocity. Detailed metric definitions and units are provided in Tables S2 and S3 in Multimedia Appendix 2.

Five themes were identified in the analysis: (1) functional requirements to interpret movement quality and performance, (2) essential aspects of movement quality, (3) added value of real-world performance, (4) individualizing what matters, and (5) blending clinical eye and reference data. Each theme comprises between 2 and 4 subthemes, as shown in Figure 2. Illustrative quotations are referenced in the text using an identifier (eg, Q1). The corresponding quotations, including participant IDs and full wording, are provided in Tables S1-S5 in Multimedia Appendix 3. Quantitative ratings of *kinematic* and *real-world performance metrics* are presented in Figure 3 and are reported convergently within respective themes.

### Theme 1: Functional Requirements to Interpret Movement Quality and Performance

This theme outlines the functional requirements that clinicians considered essential for meaningful interpretation of movement quality, whether assessed through observational movement analysis or instrumented kinematic analysis. These prerequisites, typically assessed through standard clinical evaluations, provide the baseline context needed to interpret technology-based metrics. Participants highlighted 4 key aspects as particularly decisive for understanding upper limb performance: range of motion, muscle strength, selective muscle control, and grasping function (Table S1 in Multimedia Appendix 3).

#### Range of Motion

Participants consistently emphasized that both active and passive ROM must be known to interpret movement quality and determine whether upper limb ADL can be attempted. For instance, elevation above 90° was highlighted as necessary for tasks such as combing hair or reaching overhead [Q1]. Importantly, clinicians considered functional joint interaction, such as shoulder, elbow, and spinal mobility, as a whole rather than in isolation.

#### Muscle Strength

Assessing strength was considered an important complement to active ROM, as it provides information on stability and control during functional movements such as grasping or lifting objects. Rather than isolated maximal testing, participants preferred strength assessment in task contexts, as this better reflects real-world performance. One clinician noted that strength “is always somewhere in relation to mobility” [Q2].

#### Selective Muscle Control

The ability to activate muscles selectively, such as flexing the elbow while relaxing the extensors, was described as crucial for coordinated movement. Impaired selectivity, often linked with sensory-motor deficits, could lead to compensatory strategies or coexist with abnormal muscle tone [Q3, Q4]. Clinicians highlighted that such neuromuscular control issues strongly influence how movement quality should be interpreted.

#### Grasping Function

Finally, hand opening and grip strength were identified as the most practical tests of functional capacity. Grasping integrates ROM, strength, and selectivity into a single action and was considered indispensable for ADL tasks involving objects, such as drinking from a glass [Q5].

Taken together, these 4 requirements provide a functional foundation for interpreting movement analysis in clinical contexts. Without them, technology-driven assessments risk misrepresenting patients’ true abilities. Participants also noted that this information helps them *reason about the causes of impaired movement quality*, for example, whether limitations in ROM, strength, selectivity, or grasping contribute to observed difficulties.

### Theme 2: Essential Aspects of Movement Quality

This theme highlights movement smoothness, efficiency, and compensatory movements as the 3 key attributes that clinicians use to judge movement quality in relation to the drinking task (Table S2 in Multimedia Appendix 3). These aspects capture how well movements are performed, how effortful they are, and whether patients rely on compensatory patterns.

#### Movement Smoothness

Participants referred to fluent versus jerky movement as meaningful indicators of movement quality [Q6.2]. Participants with research experience favored jerk as an intuitive single-value measure, whereas therapists were less familiar with such metrics. Although the concept of smoothness appeared familiar to therapists, they were not familiar with jerk or interjoint coordination, as reflected in ratings ranging from 4 (40%) to 5 (50%) across 10 participants (Figure 3). The *kinematic metrics* of the movement strategy domain for the drinking task were considered irrelevant or unintuitive and were not selected by any participant.

#### Movement Efficiency

Efficiency was understood as completing a task within a reasonable time and without visible strain. For instance, clinicians looked for speed and absence of effort cues, such as facial tension [Q7.1]. The metric *task duration* was indicated as important by all participants. The time required to complete a task was found to be an interesting factor in determining whether the task would be adopted into daily movement behavior. Quantitative prioritization echoed this emphasis: *task duration* received the highest mean rating among *kinematic metrics* (Figure 3).

#### Compensatory Movement

Compensatory movement, especially excessive trunk flexion or shoulder elevation, was considered a marker for adaptation to motor impairment [Q8.1]. Thus, clinicians were particularly interested in distinguishing between compensatory and physiologically correct movement. Participants found the analysis of compensatory movements challenging due to the dynamic interactions among multiple joints. Participants expected certain compensatory movement patterns involving the shoulder and torso [Q8.2, Q8.3], based on functional deficits highlighted in theme 1.

### Theme 3: Added Value of Real-World Performance

Clinicians emphasized that *what patients do outside the therapy room* ultimately determines functional recovery. Three interrelated aspects of real-world performance framed this discussion: the amount and patterns of arm use, efficiency and endurance, and contextual factors influencing ADL behavior (Table S3 in Multimedia Appendix 3).

#### Linking Capacity and Arm Use Profiles

Participants valued continuous activity profiles across the day, with timing information to link activities to mealtimes or therapy sessions. Altered patterns, such as asymmetrical arm use, were flagged as essential indicators of disability and the need for intervention [Q9.1-Q9.3]. The ability to track both the amount and timing of use was seen as highly relevant for follow-up. Quantification of the functional workspace, as reflected in terms such as “volume” or “radius” of purposeful movement, was considered highly relevant for capturing real-world performance [Q10] relative to instantaneous ROM and muscle strength [Q11].

#### Efficiency and Endurance

Clinicians emphasized that performance in everyday tasks depends not only on speed but also on the sustained capacity to repeat movements without excessive strain [Q10.1, Q10.2]. They noted that the effortless execution of a straightforward standardized task indicated a likely transfer into ADLs, whereas repeatedly eating or drinking at mealtimes required substantially more endurance due to the effortful nature of the movements [Q10.3]. This distinction between instantaneous efficiency and sustained endurance was considered central to judging functional outcomes.

#### Contextual Factors

Clinicians considered individual motivation, fatigue tolerance, and frustration levels, along with environmental influences such as assistive devices, to be crucial for real-world performance [Q11.1]. Wheelchair use was mentioned as particularly important, as it fundamentally shapes the range and types of upper limb movement available [Q11.2]. Such contextual modifiers were consistently described as necessary to interpret measures of arm-use volume and endurance in relation to each patient’s capacities and circumstances.

Taken together, clinicians considered real-world performance metrics most clinically meaningful when they combined patterns of arm use with endurance and contextual information.

### Theme 4: Individualizing What Matters

This theme underscores the importance of individualized selection of assessments, as standardized protocols often fail to capture patient-specific differences. Clinicians highlighted tailoring measures to priority ADLs, balancing functional relevance with feasibility, and adapting to diagnosis-specific challenges (Table S4 in Multimedia Appendix 3).

#### Profiling Patients

Patients with the same diagnosis often require different selective assessments depending on residual function, spasticity, or use of assistive devices. For example, transfers in paraplegic patients demand highly adaptive assessments of wheelchair transitions: One participant stated, “In our patient clientele, I would say that this is more in the direction of highly individualized functions rather than a one-size-fits-all model” [Q12.1]. As confirmed by another clinician, severely affected patients, in particular, require a problem-specific assessment in addition to standardized measurements [Q12.2]. Standardized protocols were mentioned as often not suitable, for instance, in patients with SCI, where residual function and spasms can be highly variable. Clinicians agreed that tailoring was especially important for patients with severe conditions and high support needs.

#### Therapy Relevance and Goal Setting

Goal setting and intervention, informed by individualized assessment, were other key components that emerged from expert discussions [Q13.1]. For example, *real-world performance metrics* were individually screened for patients prone to compensation or learned nonuse, and consequently, effective intervention programs, such as constraint-induced movement therapy or an individual tele-rehabilitation program [Q13.2], were selected. Participants would identify metrics indicating limitations in performing ADLs and evaluate potential changes in their real-world performance profile following the intervention. Therapy goals focus on enhancing specific functions that directly support meaningful activity improvements, with assessments tailored to measurable parameters and joints for active therapeutic intervention. Function alone is not considered a sufficient outcome measure if it is not translated into daily life activity [Q13.4].

#### Balancing Relevance and Feasibility

Active ROM and muscle strength were prioritized as the most relevant metrics, yet clinicians considered their feasibility and level of detail needed: “You also have to think about the clinic […] what can be measured in everyday clinical practice, or what is realistic here in a laboratory?” [Q14.1]. Basic information, such as whether the upper limb could be moved through full ROM against gravity, was sufficient to judge the patient’s capability. Some questioned the value of standardized impairment measures, noting that “What we measure [in] muscle status does not mean that a patient can really use it in the activity” [Q14.2]. Real-world arm use was therefore considered more informative, though more challenging to capture feasibly in daily practice.

### Theme 5: Blending Clinical Eye and Reference Data

This theme described how clinicians combine subjective observation, objective reference values, and reasoning across interacting constructs when interpreting movement analysis (Table S5 in Multimedia Appendix 3).

#### Subjective Evaluation

Clinicians agreed on the importance of visual movement analysis for primarily assessing functional impairment. Clinicians argued to “simply look” and identify how a problematic movement task begins, proceeds, and ends without formal quantification [Q15.1]. Thus, visual observation is an integral part of the therapeutic setting and a straightforward way to identify a motor problem. When exploring potential causes for abnormal movement characteristics (compensatory or jerky movement), taking a closer look at movement units and interpreting *kinematic metrics* became more interesting [Q15.4]. Despite the interest in movement quality, we identified generally cautious opinions on whether *kinematic metrics* would confirm visually flagged deficiencies or support interpretation [Q15.4].

#### Objective Reference Values

Reference values and normative data played important roles in interpretation and clinical implications. Participants expressed the need for reference values to compare patients with healthy individuals and to assess the meaningfulness of changes registered by *digital metrics of functioning*. Normative data from healthy individuals were consistently mentioned for interpreting movement smoothness metrics (eg, jerk or multijoint coordination). The comparison between the left and right upper limbs was considered a practical norm for patients with unilateral impairments such as stroke [Q16.1]. For bilateral conditions like SCI, however, clinicians emphasized that reference to healthy controls was crucial [Q16.5]. Yet even when normative data were available, interpreting *kinematic metrics* remained challenging. Because daily movement behaviors are highly variable, applying and comparing such benchmarks to real-world performance was approached with caution [Q16.6].

#### Integrating Complex Interactions

Clinicians acknowledged the difficulty of disentangling the interrelationships among strength, ROM, coordination, and sensation, which often jointly shape real-world performance. Deviations in smoothness or interjoint coordination were seen as cues to probe underlying impairments, such as force deficits, motor control issues, or sensory loss [Q17.1-Q17.7]. They also emphasized that movement quality (*kinematic metrics*) and *real-world performance metrics* are closely interrelated and that only empirical experience with these measures in practice will build the expertise needed to interpret them reliably [Q17.7]. While movement quality and real-world performance were considered central to clinical reasoning, clinicians acknowledged limited experience applying these metrics and anticipated that their value might shift as they are used more widely in clinical contexts.

## Discussion

This study examined clinicians’ views on the constructs that underpin functional health, identified which *digital metrics of functioning* were considered clinically meaningful, and outlined what is needed for intuitive integration into clinical systems. Clinicians reasoned across *functional requirements*, *kinematic* and *real-world performance metrics*, while tailoring judgments to the individual and anchoring observations to reference information. Together, these themes demonstrate how *digital metrics of functioning* can become clinically interpretable when integrated into patient-specific reasoning and contextualized within conventional assessment frameworks.

### Functional Requirements as the Foundation

With respect to objective 1, clinicians emphasized that ROM, strength, selective muscle control, and grasping function form the foundation for functional health and for the interpretation of movement analysis. Testing functional requirements (eg, passive ROM) is recommended as critical before conducting standardized observation-based assessments, such as the Fugl-Meyer Assessment for Upper Extremity (FMA-UE) [42] and the Action Research Arm Test (ARAT) [43]. The FMA-UE and ARAT are core assessments for stroke [8,11]. They also serve as a reference for identifying benchmark *kinematic* [21,44] and *real-world performance metrics* [29,45] in individuals with neurologic conditions. Notably, impairment measures and *digital metrics of functioning* are not only highly correlated but also exhibit similar recovery trajectories [46,47]. These relationships are robust yet not interchangeable: task instructions (eg, maximal speed vs comfortable speed) and compensatory movements can alter kinematic profiles without corresponding changes in impairment. The reliance on basic impairment and capacity assessment reflects long-standing rehabilitation research. Our findings reinforce that *digital metrics of functioning* should complement rather than replace these clinical assessments.

### Digital Metrics of Functioning: Relevance and Meaning

Considering objective 2, the clinicians prioritized *digital metrics of functioning* that are simple, familiar, and intuitively interpretable. In our study, the drinking task duration, number of movement units, trunk displacement, and dynamic motion trajectories of the elbow and shoulder joints were the metrics consistently selected across professions. These metrics have previously shown good reliability [48] and responsiveness (ie, sensitivity to change over time) [47] in stroke populations, and also inform observation-based assessment of drinking tasks [49]. While these metrics were evaluated in the context of the drinking task, the underlying constructs they represent, such as movement efficiency, smoothness, and compensatory strategies, are relevant across a range of functional upper limb activities. Quantifying upper limb movement quality has been found to be critical for assessing and preventing compensatory movements [38], particularly abnormal interactions among shoulder, elbow, and trunk motion trajectories [25,49,50]. Clinicians in our sample found quantification of compensatory movements or movement intermittency/jerk particularly important, as these metrics could help explain a person’s limited real-world arm use outside the therapy setting.

### Performance Outside Therapy

Revealing patients’ activity performance outside inpatient therapy sessions, or even in habitual environments, is considered highly relevant by clinicians in this and other cohorts [51-54]. This insight into real-world arm use behavior enables goal setting and evaluation. This performance-centered clinical decision-making may be enhanced when therapists are provided with sensor-based measures [51,53,55], whereas therapists showed a preference for activity capacity measures [56].

Advances in measurement technology and data processing enable differentiation of activity types, such as walking [57,58], distinguishing functional from nonfunctional upper limb movements [59,60], and even movement primitives, such as reaching or object transportation [61,62]. About half of the therapists in this study selected more specific metrics, such as reaching count and movement space, which were also reported in other studies on clinicians’ preferences [38,63]. Therapists in our study emphasized daily arm use behavior, which is effectively captured by arm-use duration metrics, including symmetry, both of which are recognized as key indicators in stroke rehabilitation research [28,29,64]. Performance duration metrics are intuitive and straightforward because they directly measure time, making them easy to understand without additional context. Notably, therapists working in rehabilitation after SCI did not consider symmetry metrics important, which lowered the overall percentage of symmetry metrics. Consistent with theme 4 (individualizing what matters), this suggests that metric relevance is shaped by diagnosis-specific reasoning: in unilateral conditions such as stroke, between-limb symmetry is a meaningful comparison, whereas in bilateral conditions such as SCI, it loses interpretive value. Selection of real-world performance metrics should therefore be aligned with the clinical population rather than applied uniformly.

### Reference and Context for Interpretation

Revealing the meaning behind naked numbers can be challenging and requires complementary information, especially for clinicians who lack experience and knowledge about metrics and their psychometric properties. With respect to objective 3, clinicians expressed a need for visual references and normative data to interpret *kinematic metrics*, as well as contextual information and normative data for *real-world performance metrics*. Daily arm use performance can be highly variable, influenced by numerous factors, including health status, activity limitations, functional impairment, personal, and environmental factors [35]. To support interpretation and informed decision-making, clinicians in this study expressed a need for population-specific reference data, alongside cutoff values (eg, percentiles, visual benchmarks, or expected recovery trajectories), to determine whether observed changes are clinically meaningful. In clinical settings, aligning data with daytime and therapy schedules is a practical way to contextualize performance metrics to activity type [51,65]. For goal setting and evaluating therapy effectiveness, the minimal detectable change (MDC) and minimal important change (MIC) are important references for within-person change. Consequently, the successful integration of *digital metrics of functioning* into clinical reasoning depends on their linkage to patient-specific contexts, diagnostic-specific expectations, and usable normative data to inform treatment planning and progression. Benchmarking schemes for upper limb *kinematic* and *real-world performance metrics* [66], together with patient-centered benchmarking approaches [67], offer concrete frameworks that translate changes in *digital metrics of functioning* into actionable targets for neurorehabilitation practice.

### Future Directions

Electronic health records are the backbone of information collection and sharing among clinical professionals. An integration of continuous monitoring of *digital metrics of functioning* could enable personalized rehabilitation, provide richer representations of a patient’s health status, and enable timely detection of recovery plateaus or declines in real-world performance. To ensure clinical interpretability across heterogeneous neurorehabilitation populations, robust reference datasets are needed that include healthy controls and relevant patient groups, stratified by diagnosis, age, hand dominance, and impairment level. For *kinematic metrics*, task-specific benchmarks for validated tasks, such as the drinking tasks [25,48,68], could provide z-scores or percentiles alongside MDC/MIC thresholds. For *real-world performance metrics*, normative data and MIC thresholds exist but remain limited to stroke populations [29].

Future research should explore the optimal convergent set of meaningful, intuitive information that is accessible through graphical illustration. Nearly a decade ago, Ploderer et al [69] proposed dashboard designs that bring together ROM, kinematic analyses, hourly activity patterns, and 3D functional workspace visualizations. The need for integrated, actionable displays remains, but infrastructural and implementation barriers continue to slow adoption [70,71]. Currently, measurement technologies that capture upper limb *digital metrics of functioning*, such as 3D trajectory analysis and ADL movement space and task detection, remain largely impractical for routine clinical settings. Advances in scalable sensor- or computer vision-based systems are therefore crucial for facilitating precise, clinically relevant visualizations of *kinematic metrics* and functional reaching space during ADL.

Building on the identified clinical reasoning priorities, Weikert et al [72] developed a learning health system embedded in clinical neurorehabilitation practice. This integrated learning health system enables an end-to-end evaluation of multimodal patient data capture and clinical data presentation. These systems aim to integrate kinematics [73], performance data, cognitive/mental status, patient-reported outcomes, speech, sleep, nutrition, and contextual information (including therapy schedules and temporal patterns) [72]. A key principle for clinical embedding is that clinician-facing outcomes must provide meaningful information to ensure adherence and acceptance.

Such systems directly address two priorities raised by our participants: blending objective metrics with contextual reference information (theme 5) and combining task-based capacity with real-world performance profiles (theme 3). Realizing this potential, however, requires systematic evaluation of the system components, the clinical utility [74], usability [75,76], and the real-world effectiveness of new digital outcome domains. Ultimately, a multipronged approach comprising clinical value systems [77], educational programs, and user-friendly data capture is needed to introduce precision neurorehabilitation at scale [78-80].

Training programs should align with recognized digital-health competency frameworks [81,82] to ensure clinicians acquire the knowledge, skills, and attitudes needed to interpret and act upon *digital metrics of functioning*. As demonstrated in electronic health record–based educational initiatives, targeted modules on dashboard use and metric literacy can improve clinician proficiency in real-world data-driven workflows [83]. In the present context, educational efforts should therefore focus on foundational metric interpretation and practical integration of *digital metrics of functioning* into routine decision-making. Such programs could serve as an educational scaffold for translating digital metric evidence into individualized, data-informed rehabilitation planning.

### Limitations

This study was conducted in 3 Swiss rehabilitation clinics, which may limit transferability to other health care systems, settings (inpatient vs. outpatient), and languages/cultures. The low participation rate (3/8 clinics, 38%) was primarily due to organizational constraints in releasing multiple clinicians simultaneously for a 90-minute focus group discussion. Recruitment through clinic nomination may have favored clinicians who are more open to digital assessments, potentially more optimistic in their perspectives, although skeptical views also emerged during the focus groups.

The small number of focus groups and the limited number of participants per group may have influenced group interaction dynamics and the breadth of perspectives discussed. Given the limited number and size of focus groups, thematic saturation cannot be assumed. Furthermore, the small group sizes and site-specific sampling limit the transferability of the findings to clinics with a broader mix of patient populations. Consistent with the exploratory qualitative design, we aimed to provide information-rich insights rather than comprehensive coverage. Accordingly, our findings should be interpreted as exploratory and context-dependent rather than representative or generalizable.

The concept of “relevance” was not operationalized using predefined criteria during the focus group discussions. This approach was chosen to allow clinicians to articulate their own interpretations and reasoning processes. However, participants may have interpreted the term differently, for example, in terms of clinical usefulness, familiarity, feasibility, or patient-centeredness. Such variability may introduce construct ambiguity or response-process bias. While the qualitative analysis allowed us to capture these perspectives through thematic interpretation, future studies could benefit from more explicit operationalization or structured rating criteria.

In addition, the variation in clinicians’ prior experience with *digital metrics of functioning* may have influenced interpretations. Future research should examine how perspectives differ across professional roles, experience levels, and clinical contexts and assess the impact of targeted training and exposure on the interpretation of *digital metrics of functioning* in clinical routine.

Moreover, participants were shown an example of a patient performing a drinking task and were evaluated using kinematic metrics validated in this context to provide a tangible, standardized reference and ensure a common frame of reference. While this approach supported understanding of abstract kinematic concepts, presenting a specific task and task-validated metrics may have introduced contextual bias and may limit the full generalizability of the findings.

Both moderator and co-moderator were clinician-researchers, introducing potential social desirability bias despite reflexive views. To mitigate this risk, analytic decisions and theme development were documented through saved versions of code structures, cluster maps, and theme definitions, allowing us to track the evolution of codes and themes.

Finally, we focused on the clinical significance of *digital metrics of functioning* while intentionally setting aside practical constraints. The feasibility, usability, and data-quality requirements of instrumented assessments (eg, sensor setup and maintenance, nonwear detection, calibration procedures, processing resources, and data privacy demands) warrant dedicated evaluation before routine deployment.

### Conclusions

This exploratory study mapped how clinicians in Swiss neurorehabilitation interpret and prioritize digital metrics of functioning of the upper limb. Participants valued *kinematic* and *real-world performance* metrics when they were embedded in patient-specific reasoning and paired with contextual information. They consistently favored intuitive metrics, such as task duration, number of movement units, and ROM, over more complex measures whose interpretation requires specialized knowledge. Across the five themes, three priorities emerged for translating digital metrics of functioning into routine practice: (1) stratified normative and reference datasets and meaningful change thresholds, integrated into co-designed dashboards that link task-based kinematic metrics to daily activity profiles; (2) targeted training that builds metric literacy and supports interpretation alongside conventional clinical reasoning; and (3) scalable measurement technologies that integrate seamlessly into clinical workflows, evaluated pragmatically for decision impact, usability, and adoption. These findings should be interpreted as context-dependent rather than generalizable, given the small number of focus groups and the specific clinical and organizational settings sampled. Perspectives are likely to vary across health care systems, professional backgrounds, and levels of experience with digital assessments. Nonetheless, the convergence across professions and clinics on the need for interpretability, contextualization, and clinical embedding offers a clear direction for future work. Centering on clinician and patient preferences will be essential for meaningful integration of *digital metrics of functioning* into clinical practice and for narrowing the evidence-practice gap.

## Supplementary material

10.2196/87339 Multimedia Appendix 1 Interview guideline for focus groups.

10.2196/87339 Multimedia Appendix 2 Ratings of ICF parameters and digital metrics of functioning. ICF: International Classification of Functioning, Disability and Health.

10.2196/87339 Multimedia Appendix 3 Participants' quotations per theme and subtheme.

10.2196/87339 Checklist 1 SRQR checklist.

## Abbreviations

ADL: activities of daily living
ARAT: Action Research Arm Test
FMA-UE: Fugl-Meyer Assessment for Upper Extremity
HRA: Swiss Federal Act on Research involving Human Beings
ICF: International Classification of Functioning, Disability and Health
MDC: minimal detectable change
MIC: minimal important change
ROM: range of motion
SCI: spinal cord injury
SRQR: Standards for Reporting Qualitative Research

## References

[1] Javeed S, Greenberg JK, Zhang JK (2023). Association of upper-limb neurological recovery with functional outcomes in high cervical spinal cord injury. J Neurosurg.

[2] Lawrence ES, Coshall C, Dundas R (2001). Estimates of the prevalence of acute stroke impairments and disability in a multiethnic population. Stroke.

[3] (2001). International Classification of Functioning, Disability and Health (ICF). World Health Organization.

[4] ten Cate O, ten Cate O, Custers E, Durning S (2018). Principles and Practice of Case-Based Clinical Reasoning Education.

[5] Brogioli M, Schneider S, Popp WL (2016). Monitoring upper limb recovery after cervical spinal cord injury: insights beyond assessment scores. Front Neurol.

[6] Kwakkel G, Kollen B, Lindeman E (2004). Understanding the pattern of functional recovery after stroke: facts and theories. Restor Neurol Neurosci.

[7] Kwakkel G, Stinear C, Essers B (2023). Motor rehabilitation after stroke: European Stroke Organisation (ESO) consensus-based definition and guiding framework. Eur Stroke J.

[8] Pohl J, Held JPO, Verheyden G (2020). Consensus-based core set of outcome measures for clinical motor rehabilitation after stroke—a Delphi study. Front Neurol.

[9] Kwakkel G, Lannin NA, Borschmann K (2017). Standardized measurement of sensorimotor recovery in stroke trials: consensus-based core recommendations from the Stroke Recovery and Rehabilitation Roundtable. Int J Stroke.

[10] Demers M, Levin MF (2017). Do activity level outcome measures commonly used in neurological practice assess upper-limb movement quality?. Neurorehabil Neural Repair.

[11] Kwakkel G, Van Wegen E, Burridge JH (2019). Standardized measurement of quality of upper limb movement after stroke: consensus-based core recommendations from the Second Stroke Recovery and Rehabilitation Roundtable. Int J Stroke.

[12] Essers B, Meyer S, De Bruyn N (2019). Mismatch between observed and perceived upper limb function: an eye-catching phenomenon after stroke. Disabil Rehabil.

[13] van Delden A, Peper CLE, Beek PJ, Kwakkel G (2013). Match and mismatch between objective and subjective improvements in upper limb function after stroke. Disabil Rehabil.

[14] Doman CA, Waddell KJ, Bailey RR, Moore JL, Lang CE (2016). Changes in upper-extremity functional capacity and daily performance during outpatient occupational therapy for people with stroke. Am J Occup Ther.

[15] Lang CE, Holleran CL, Strube MJ (2023). Improvement in the capacity for activity versus improvement in performance of activity in daily life during outpatient rehabilitation. J Neurol Phys Ther.

[16] Levin MF, Kleim JA, Wolf SL (2009). What do motor “recovery” and “compensation” mean in patients following stroke?. Neurorehabil Neural Repair.

[17] Kanzler CM, Lamers I, Feys P, Gassert R, Lambercy O (2021). Personalized prediction of rehabilitation outcomes in multiple sclerosis: a proof-of-concept using clinical data, digital health metrics, and machine learning. Med Biol Eng Comput.

[18] Schwarz A, Bhagubai MMC, Nies SHG (2022). Characterization of stroke-related upper limb motor impairments across various upper limb activities by use of kinematic core set measures. J NeuroEngineering Rehabil.

[19] Rand D, Eng JJ (2012). Disparity between functional recovery and daily use of the upper and lower extremities during subacute stroke rehabilitation. Neurorehabil Neural Repair.

[20] Essers B, Veerbeek JM, Luft AR, Verheyden G (2024). The feasibility of the adapted H-GRASP program for perceived and actual daily-life upper limb activity in the chronic phase post-stroke. Disabil Rehabil.

[21] Schwarz A, Kanzler CM, Lambercy O, Luft AR, Veerbeek JM (2019). Systematic review on kinematic assessments of upper limb movements after stroke. Stroke.

[22] Kalsi-Ryan S, Beaton D, Curt A, Popovic MR, Verrier MC, Fehlings MG (2014). Outcome of the upper limb in cervical spinal cord injury: profiles of recovery and insights for clinical studies. J Spinal Cord Med.

[23] Rudd KD, Lawler K, Callisaya ML, Alty J (2023). Investigating the associations between upper limb motor function and cognitive impairment: a scoping review. Geroscience.

[24] Alt Murphy M, Willén C, Sunnerhagen KS (2012). Movement kinematics during a drinking task are associated with the activity capacity level after stroke. Neurorehabil Neural Repair.

[25] Alt Murphy M, Murphy S, Persson HC, Bergström UB, Sunnerhagen KS (2018). Kinematic analysis using 3D motion capture of drinking task in people with and without upper-extremity impairments. J Vis Exp.

[26] Lili L, Sunnerhagen KS, Rekand T, Alt Murphy M (2021). Quantifying an upper extremity everyday task with 3D kinematic analysis in people with spinal cord injury and non-disabled controls. Front Neurol.

[27] Kim GJ, Parnandi A, Eva S, Schambra H (2021). The use of wearable sensors to assess and treat the upper extremity after stroke: a scoping review. Disabil Rehabil.

[28] Barth J, Lohse KR, Konrad JD, Bland MD, Lang CE (2021). Sensor-based categorization of upper limb performance in daily life of persons with and without neurological upper limb deficits. Front Rehabil Sci.

[29] Pohl J, Verheyden G, Held JPO, Luft AR, Easthope Awai C, Veerbeek JM (2025). Construct validity and responsiveness of clinical upper limb measures and sensor-based arm use within the first year after stroke: a longitudinal cohort study. J Neuroeng Rehabil.

[30] Braakhuis HEM, Bussmann JBJ, Ribbers GM, Berger MAM (2021). Wearable activity monitoring in day-to-day stroke care: a promising tool but not widely used. Sensors (Basel).

[31] Yang CL, Cheng YY, Lin CH, Menon C, Eng JJ (2025). Quantifying the reach-and-grasp practice using novel wearable technology: application in a stroke rehabilitation setting. J Hand Ther.

[32] Okuyama K, Kawakami M, Tsuchimoto S (2020). Depth sensor-based assessment of reachable work space for visualizing and quantifying paretic upper extremity motor function in people with stroke. Phys Ther.

[33] Gomes CLA, Cacho RO, Nobrega VTB (2021). Effects of attentional focus on upper extremity motor performance in post stroke patients: a randomized pilot study. Medicine (Baltimore).

[34] Wu C yi, Trombly CA, Lin K chung, Tickle-Degnen L (2000). A kinematic study of contextual effects on reaching performance in persons with and without stroke: influences of object availability. Arch Phys Med Rehabil.

[35] Gagné-Pelletier L, Poitras I, Roig M, Mercier C (2025). Factors associated with upper extremity use after stroke: a scoping review of accelerometry studies. J Neuroeng Rehabil.

[36] Tausch AP, Menold N (2016). Methodological aspects of focus groups in health research. Glob Qual Nurs Res.

[37] Wilkinson S (1998). Focus groups in health research. J Health Psychol.

[38] Simpson LA, Menon C, Hodgson AJ, Ben Mortenson W, Eng JJ (2021). Clinicians’ perceptions of a potential wearable device for capturing upper limb activity post-stroke: a qualitative focus group study. J Neuroeng Rehabil.

[39] Braun V, Clarke V (2006). Using thematic analysis in psychology. Qual Res Psychol.

[40] Rampin R, Rampin V (2021). Taguette: open-source qualitative data analysis. J Open Source Soft.

[41] O’Brien BC, Harris IB, Beckman TJ, Reed DA, Cook DA (2014). Standards for reporting qualitative research. Acad Med.

[42] Hervé-Colas J, Newton SP, Engelter ST (2026). Standardized international manual of the Fugl-Meyer assessment of motor function after stroke. Neurorehabil Neural Repair.

[43] Yozbatiran N, Der-Yeghiaian L, Cramer SC (2008). A standardized approach to performing the Action Research Arm Test. Neurorehabil Neural Repair.

[44] Kanzler CM, Rinderknecht MD, Schwarz A (2020). A data-driven framework for selecting and validating digital health metrics: use-case in neurological sensorimotor impairments. NPJ Digit Med.

[45] Lohse KR, Miller AE, Bland MD, Lee JM, Lang CE (2024). Validation of real-world actigraphy to capture post-stroke motor recovery. medRxiv.

[46] Lang CE, Waddell KJ, Barth J, Holleran CL, Strube MJ, Bland MD (2021). Upper limb performance in daily life approaches plateau around three to six weeks post-stroke. Neurorehabil Neural Repair.

[47] Thrane G, Sunnerhagen KS, Murphy MA (2020). Upper limb kinematics during the first year after stroke: the stroke arm longitudinal study at the University of Gothenburg (SALGOT). J Neuroeng Rehabil.

[48] Frykberg GE, Grip H, Alt Murphy M (2021). How many trials are needed in kinematic analysis of reach-to-grasp? A study of the drinking task in persons with stroke and non-disabled controls. J Neuroeng Rehabil.

[49] Jose M, Munoz-Novoa M, Alt Murphy M (2024). A reliable and valid assessment of upper limb movement quality after stroke: the observational drinking task assessment. J Rehabil Med.

[50] Sauerzopf L, Panduro CGC, Luft AR (2024). Evaluating inter- and intra-rater reliability in assessing upper limb compensatory movements post-stroke: creating a ground truth through video analysis?. J Neuroeng Rehabil.

[51] Jung HT, Kim Y, Lee J, Lee SI, Choe EK (2022). Envisioning the use of in-situ arm movement data in stroke rehabilitation: stroke survivors’ and occupational therapists’ perspectives. PLoS ONE.

[52] Mayrhuber L, Roffler L, Easthope CA, Gassert R, Lambercy O (2025). 2025 International Conference on Rehabilitation Robotics (ICORR).

[53] Miller AE, Holleran CL, Bland MD (2024). Perspectives of key stakeholders on integrating wearable sensor technology into rehabilitation care: a mixed-methods analysis. medRxiv.

[54] Serrano LP, Maita KC, Avila FR (2023). Benefits and challenges of remote patient monitoring as perceived by health care practitioners: a systematic review. Perm J.

[55] Holleran CL, Bland MD, Lang CE (2023). Comprehensive assessment of the activity level of the ICF using both capacity and performance measures: a case report. Arch Rehabil Res Clin Transl.

[56] Bland MD, Holleran CL, Newman CA (2024). ICF classification of therapeutic goals for outpatient episodes of neurorehabilitation in post-stroke and Parkinson disease. Disabil Rehabil.

[57] Leuenberger KD (2015). Long-term activity and movement monitoring in neurological patients. Thesis.

[58] Pohl J, Ryser A, Veerbeek JM (2022). Accuracy of gait and posture classification using movement sensors in individuals with mobility impairment after stroke. Front Physiol.

[59] Lum PS, Shu L, Bochniewicz EM (2020). Improving accelerometry-based measurement of functional use of the upper extremity after stroke: machine learning versus counts threshold method. Neurorehabil Neural Repair.

[60] Pohl J, Ryser A, Veerbeek JM (2022). Classification of functional and non-functional arm use by inertial measurement units in individuals with upper limb impairment after stroke. Front Physiol.

[61] Kaku A, Parnandi A, Venkatesan A, Pandit N, Schambra H, Fernandez-Granda C (2020). Towards data-driven stroke rehabilitation via wearable sensors and deep learning. Proc Mach Learn Res.

[62] Parnandi A, Kaku A, Venkatesan A (2023). Data-driven quantitation of movement abnormality after stroke. Bioengineering (Basel).

[63] van Meulen FB, Reenalda J, Buurke JH, Veltink PH (2014). Assessment of daily-life reaching performance after stroke. Ann Biomed Eng.

[64] Macpherson CE, Bland MD, Gordon C (2025). Replication of sensor-based categorization of upper-limb performance in daily life in people post stroke and generalizability to other populations. Sensors (Basel).

[65] McLaren R, Signal N, Lord S, Taylor S, Henderson J, Taylor D (2019). The volume and timing of upper limb movement in acute stroke rehabilitation: still room for improvement. Disabil Rehabil.

[66] Longatelli V, Torricelli D, Tornero J (2022). A unified scheme for the benchmarking of upper limb functions in neurological disorders. J Neuroeng Rehabil.

[67] Manta C, Patrick-Lake B, Goldsack JC (2020). Digital measures that matter to patients: a framework to guide the selection and development of digital measures of health. Digit Biomark.

[68] Unger T, de Sousa Ribeiro R, Mokni M (2024). Upper limb movement quality measures: comparing IMUs and optical motion capture in stroke patients performing a drinking task. Front Digit Health.

[69] Ploderer B, Fong J, Klaic M (2016). How therapists use visualizations of upper limb movement information from stroke patients: a qualitative study with simulated information. JMIR Rehabil Assist Technol.

[70] Cain A, Gunby T, Winstein C, Demers M (2025). Advancing stroke rehabilitation: the role of wearable technology according to research experts. Disabil Rehabil Assist Technol.

[71] Swain TA, McNarry MA, Mackintosh KA (2024). Assessing and enhancing movement quality using wearables and consumer technologies: thematic analysis of expert perspectives. JMIR Form Res.

[72] Weikert T, Paez-Granados D, Stoller O, Easthope CA (2024). Converging Clinical and Engineering Research on Neurorehabilitation.

[73] Unger T, Moslehian AS, Peiffer JD (2024). Differentiable biomechanics for markerless motion capture in upper limb stroke rehabilitation: a comparison with optical motion capture 2024. arXiv.

[74] Weikert T, Li Y, Paez-Granados D, Easthope CA (2025). 2025 International Conference on Rehabilitation Robotics (ICORR).

[75] Du E, D. Peiffer J, Weikert T, Awai CE, James Cotton R (2025). Usability of iGait@Healthcore: gait analysis from a smartphone for use in clinical routine. Gait Posture.

[76] Unger T, Yao X, Schmitt A (2025). Usability of capturing upper limb movement kinematics in clinical routine: do wearable sensors beat markerless motion capture?. Gait Posture.

[77] Domnik N, Krasnopolska K, Sylvester R (2025). User-centered development of a digital platform to provide neurorehabilitation patients with feedback on health data. JMIR Preprints.

[78] Azodo I, Williams R, Sheikh A, Cresswell K (2020). Opportunities and challenges surrounding the use of data from wearable sensor devices in health care: qualitative interview study. J Med Internet Res.

[79] Denny JC, Collins FS (2021). Precision medicine in 2030—seven ways to transform healthcare. Cell.

[80] Helminski D, Sussman JB, Pfeiffer PN (2024). Development, implementation, and evaluation methods for dashboards in health care: scoping review. JMIR Med Inform.

[81] Car J, Ong QC, Erlikh Fox T (2025). The digital health competencies in medical education framework. JAMA Netw Open.

[82] Lawrence K, Levine DL (2024). The digital determinants of health: a guide for competency development in digital care delivery for health professions trainees. JMIR Med Educ.

[83] Bhavaraju VL, Panchanathan S, Willis BC, Garcia-Filion P (2024). Leveraging the electronic health record to measure resident clinical experiences and identify training gaps: development and usability study. JMIR Med Educ.

