# Correction: From Metrics to Meaning in Neurological Rehabilitation: Clinicians’ Perspectives on Digital Metrics of Upper Limb Functioning—A Focus Group Study

**DOI:** 10.2196/106631

**Published:** 2026-09-11

**Authors:** Johannes Pohl, Laura Mayrhuber, Olivier Lambercy, Chris Easthope Awai

**Affiliations:** 1 Data Analytics and Rehabilitation Technology (DART), Lake Lucerne Institute, Ruzbistrasse 9, Vitznau, 6354, Switzerland, 41 0774868083; 2 Rehabilitation Engineering Laboratory (RELab), Department of Health Sciences and Technology, ETH Zurich, Zürich, Switzerland

In “From Metrics to Meaning in Neurological Rehabilitation: Clinicians’ Perspectives on Digital Metrics of Upper Limb Functioning—A Focus Group Study” [1], the authors made one addition.

Authors JP and LM contributed equally and share first authorship. This has been specified via an equal contributor footnote.

The correction will appear in the online version of the paper on the JMIR Publications website together with the publication of this correction notice. Because this was made after submission to PubMed, PubMed Central, and other full-text repositories, the corrected article has also been resubmitted to those repositories.
