# Supplementary material for: From Metrics to Meaning in Neurological Rehabilitation: Clinicians’ Perspectives on Digital Metrics of Upper Limb Functioning—A Focus Group Study
Source: JMIR Rehabil Assist Technol. 2026 Jun 24;13:e87339. doi: 10.2196/87339 (PMC13293569; doi:10.2196/87339)
Supplement: Multimedia Appendix 1 [file rehab-v13-e87339-s001.pdf]

Interview guideline:

Focus Group discussion guide – Upper Limb Outcome (IMPACT Project)

Time schedule and allocation (90 minutes)

Duration	Content
5’	1. Introduction & consent
15-20’	2. ICF-function (Warm-Up)
25-30’	3. Movement quality
25-30’	4. ADL performance
5’	5. Wrap-up & conclusions

Introduction – before start of audio recording (15 minutes)

- Explanation of data protection and anonymization process
- Stating etiquette regarding respectful communication

Standardized introductory text:

Hello, everyone, my name is [Moderator], and I will be facilitating today's discussion.

Thank you for contributing your thoughts and taking part in this focus group.
The purpose of this session is to explore which kinematic information is crucial to form an objective understanding of upper limb function and activity. Our goal is to reach a consensus on essential parameters and how they should be visualized to support clinical reasoning processes.

There will be questions and tasks throughout the discussion. There are no ‘right’ or ‘wrong’ answers—we are interested in your professional opinions and experiences.
We value your perspectives as rehabilitation experts, and a respectful, appreciative tone is expected throughout.

The discussion will last about 60 minutes and will be audio-recorded for later analysis.
Afterwards, all data will be anonymized and cannot be traced back to individuals.

Data protection statements:

- Confidentiality: *Your participation is confidential. No personal data will be shared without your consent.*
- Data collection: *We will gather insights related to our research questions (e.g., opinions, experiences).*
- Recording: *The discussion will be recorded via audio and field notes, exclusively for research purposes.*
- Voluntary participation: *You may withdraw at any time or decline to be recorded.*
  - Consent: *By participating, you agree to these terms. If you have concerns, please let me know*.
- *Any questions before we begin recording?* → Start of recording

Introduction of participants and moderators:

Let's start with brief introductions. Please say your first name and share a short statement about your expectations for this focus group.

1. Brainstorming – *ICF* (International Classification of Functioning, Disability and Health)

- Introduction and clarification of the ICF concept

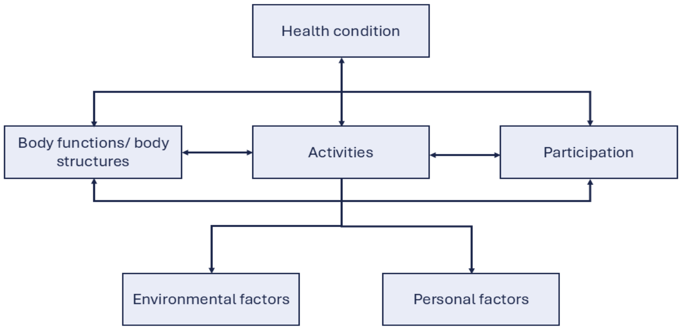

a) Brainstorming question: Which domains/characteristics do you consider important to quantify the patient's functional health condition?

➔ Map measures on a whiteboard, sorted by ICF domains

b) Which upper limb functions, such as range of motion and force components, etc., do you consider important for your typical patients?

-> Prioritize within a list, argument ROM, force components by joints, sensation, etc.

3. Discussion on kinematic and real-world performance parameters) **Movement quality (MQ) in a standardized movement (e.g., reach-to-target)**

Presentation case vignette with a video clip of a standardized drinking task performed with each upper limb separately (frontal & sagittal perspective).

Open discussion on movement quality and kinematic metrics
- Which movement characteristics would you assess or quantify?
- Why are these features clinically relevant?
- What needs to be considered when interpreting these characteristics (e.g., interdependencies)?

Prioritization of kinematic metrics
→ Hand out a list of kinematic parameters
→ Mark key parameters
→ Discuss opinions/agreement

b) Real-world performance metrics
Presentation case vignette with a video clip of the same patient drinking from a glass in an unsupervised natural context (frontal & sagittal perspective).

Open discussion on real-world performance
- Which aspects of daily upper limb performance would you quantify?
- Which usage patterns are of particular interest?
- Which contextual information is needed?

Prioritization of real-world performance parameters
→ Hand out a list of real-world performance parameters
→ Mark key parameters
→ Discuss opinions/agreement

4. Wrap up
- Summary of parts movement quality and performance

Confirm agreements:
Summing up, we can conclude that…
→ Highlight key domains: ICF, kinematics, real-world performance

Finishing statements:
- *Is there anything important we haven't yet discussed?*
- *Is everyone satisfied with the outcomes, or are there any final remarks?*

We will transcribe and analyze the data collected in the coming weeks and plan further steps accordingly. We're happy to keep you informed.
Once again, many thanks for your valuable time and contribution to this focus group.
