# Supplementary material for: From Metrics to Meaning in Neurological Rehabilitation: Clinicians’ Perspectives on Digital Metrics of Upper Limb Functioning—A Focus Group Study
Source: JMIR Rehabil Assist Technol. 2026 Jun 24;13:e87339. doi: 10.2196/87339 (PMC13293569; doi:10.2196/87339)
Supplement: Multimedia Appendix 2 [file rehab-v13-e87339-s002.pdf]

Table S1 Rating of International Classification of Functioning, Disability and Health - function parameters

ROM (active and passive)		Prioritization				Muscle strength (MFK 1-6)	Prioritization			
**Joint**	**Movement direction**	**C1 (n)**	**C2 (n)**	**C3 (n)**	**Total**	**Movement direction**	**C1 (n)**	**C2 (n)**	**C3 (n)**	**Total**
Shoulder	Elevation/ Depression	*n.a.*	*2*	*0*	*2*	Elevation/ Depression	*n.a.*	*0*	*0*	*0*
	Flexion/ Extension	*n.a.*	*0*	*3*	*3*	Flexion/ Extension	*n.a.*	*2*	*1*	*3*
	Abduction/ Adduction	*n.a.*	*0*	*0*	*0*	Abduction/ Adduction	*n.a.*	*0*	*0*	*0*
	Internal/ External rotation	*n.a.*	*0*	*1*	*1*	Internal/ External rotation	*n.a.*	*0*	*0*	*0*
Elbow	Flexion/ Extension	*n.a.*	*0*	*2*	*2*	Flexion/ Extension	*n.a.*	*2*	*0*	*0*
	Pronation/ Supination	*n.a.*	*0*	*2*	*2*	Pronation/ Supination	*n.a.*	*0*	*0*	*0*
Wrist	Flexion/ Extension	*n.a.*	*2*	*1*	*3*	Flexion/ Extension	*n.a.*	*1*	*0*	*1*
	Abduction/ Adduction	*n.a.*	*0*	*0*	*0*	Abduction/ Adduction	*n.a.*	*0*	*0*	*0*
Handgrip	Pinch grip	*n.a.*	*0*	*1*	*1*	Pinch grip	*n.a.*	*1*	*0*	*1*
	Key grip	*n.a.*	*0*	*0*	*0*	Key grip	*n.a.*	*1*	*0*	*1*
Hand - global	Grip strength	*n.a.*	*1*	*2*	*3*	Grip strength	*n.a.*	*3*	*1*	*4*
	Hand extension	*n.a.*	*1*	*2*	*3*	Hand extension	*n.a.*	*1*	*0*	*1*
Fine motor skills	Individuation	*n.a.*	*1*	*0*	*1*	Individuation	*n.a.*	*1*	*0*	*1*

Somatosensory function	Prioritization			
Pain	*n.a.*	*0*	*1*	*1*
Sensation	*n.a.*	*0*	*0*	*0*
Proprioception	*n.a.*	*0*	*2*	*2*

Prioritization of maximally five parameters by participants (n=9) from three rehabilitation centers *(C1, C2, C3)*. Participants from clinic 1 were not included in this process, but indicated their priority during the interview. *Abbreviations: n.a., not assessed; n, number of indications; ROM, range of motion; MFK, manual muscle function test.*

Table S2 Rating results and description of kinematic metrics by the rehabilitation clinic

**Kinematic metrics**		**Prioritization**			
**Domain**	**Metric**	**C1 (n)**	**C2 (n)**	**C3 (n)**	**Total (%)**
Velocity/ Movement time	Task duration (sec)	*3*	*4*	*4*	***100***
	Peak wrist velocity (m/sec)	*1*	*0*	*0*	***9***
	Peak angular velocity elbow (°)	*3*	*0*	*0*	***27***
	Peak angular velocity shoulder (°)	*0*	*0*	*0*	***0***
Smoothness/ Coordination	Number of movement units/ tries	*1*	*2*	*4*	***70***
	Inter-joint coordination (shoulder vs. elbow)	*1*	*1*	*3*	***50***
	Jerk	*0*	*3*	*1*	***40***
	Synergies (multi-joint)	*0*	*1*	*2*	***30***
Movement strategy	Time to maximal velocity (sec)	*0*	*0*	*0*	***0***
	Time to maximal velocity (% of movement)	*0*	*0*	*0*	***0***
	Time to first peak velocity (sec)	*0*	*0*	*0*	***0***
	Time to first peak velocity (%of movement)	*0*	*0*	*0*	***0***
Range of motion	Trunk displacement (mm, °)	*2*	*4*	*1*	***70***
	Shoulder flexion (°)	*0*	*4*	*4*	***80***
	Elbow extension (°)	*2*	*3*	*3*	***80***
	Shoulder abduction (°)	*2*	*3*	*2*	***70***
	Shoulder external rotation (°)	*0*	*3*	*4*	***70***

Additional information on the meaning of metrics was given upon participants request, e.g. task duration: duration from start to end of the movement; jerk: movement fluency. Abbreviations: °, degrees; m, meter; mm, millimeter; n, number of indications; sec, second.

Table S3 Rating results and description of real-world performance metrics by the rehabilitation clinic

**Real-world performance metrics**		**Prioritization**			
**Domain**	**Metrics**	**C1 (n)**	**C2 (n)**	**C3 (n)**	**Total (%)**
Duration of activity	Total duration of arm use (bilateral + unilateral)	*2*	*2*	*3*	***70***
	Bilateral duration of arm use	*1*	*4*	*2*	***70***
	Unilateral duration of arm use (left, right)	*1*	*4*	*2*	***70***
	Symmetry duration of arm use (time: affected/less affected)	*1*	*0*	*3*	***40***
Intensity of activity	Total intensity of arm use (bilateral + unilateral)	*2*	*1*	*1*	***40***
	Bilateral intensity of arm use	*1*	*1*	*2*	***40***
	Unilateral intensity of arm use (left, right)	*0*	*2*	*0*	***20***
	Symmetry intensity of arm use (time: affected/less affected)	*1*	*1*	*2*	***40***
Reaching count and space	Reaching count of movements (grasp, hand to mouth)	*0*	*2*	*3*	***50***
	Movement space (volume and degrees of freedom)	*0*	*3*	*3*	***60***

Additional information on the meaning of metrics was explained upon participants' request, e.g., unilateral duration/intensity: one-sided activity while the other side is inactive, symmetry: average balance between left and right activity. Abbreviations: n, number of indications.
