# Supplementary material for: From Metrics to Meaning in Neurological Rehabilitation: Clinicians’ Perspectives on Digital Metrics of Upper Limb Functioning—A Focus Group Study
Source: JMIR Rehabil Assist Technol. 2026 Jun 24;13:e87339. doi: 10.2196/87339 (PMC13293569; doi:10.2196/87339)
Supplement: Multimedia Appendix 3 [file rehab-v13-e87339-s003.pdf]

Participant quotations by themes and subthemes

Table S1 Quotations of Theme 1: *Requirements for functional movement*

Subtheme	ID	Quote
Active and passive ROM	Q1	*"The [capacity] setting has to be right, so that they (patients) achieve a certain range [of motion], for example [...], over 90 degrees [of shoulder flexion]. That must actually be the case." [P3-C1]*
Muscle strength	Q2	*"I put my [priorities] rather in the [muscle] strength area, because I thought strength is, if we look at the MRC [Medical Research Council] criteria, always somewhere in relation to mobility as well." [P9-C3]*
Selectivity and muscle tone	Q3	*"How selectively can [the patient] move? How much does [the patient] compensate for? How well can [the patient] activate [the muscles] selectively?" [P4-C2]*
	Q4	*"If I can provoke a spasm, then I have much more force on a force meter than if it is arbitrary. [...] Do I have spasticity or spasms at all, because that will definitely [...] influence my result." [P11-C3]*
Grasping function	Q5	*"To grasp something, finger extension is important [...], but then in the grip, ultimately, the strength of the [finger] flexion that I can hold an object" [P11-C3]*

Table S2 Quotations of Theme 2: *Critical aspects of movement quality*

Subtheme	ID	Quote
Movement smoothness	Q6.1	*"What I would like to know most is the quality of the movement when this activity is carried out in a very specific way. How does it change over time, for example?" [P9-C3]*
	Q6.2	*"If you had a very bumpy movement, this would influence the jerk [metric]. [I would choose the metric] simply for the reason, [...] because I know that you can simply measure [jerk]. And have the feeling that it also gives a first statement, before getting a global understanding of [...] how well coordinated the movement is, while the other things [multi-joint coordination] are certainly more complex, and also [more complex] to collect [the metrics] in an everyday setting." [P9-C3]*
Movement efficiency	Q7.1	*"Then also at speed. [The patient] reaches [the] goal in a reasonable time, and [they] don't seem to be straining [their] face ." [P7-C2]*
	Q7.2.	*"And the movement is specifically for a purpose or a goal? And then for me, it [movement quality] is linked to an efficient activity." [P6-C2]*
Compensatory movements	Q8.1	*"Depending on the activity and other conditions, you need certain movements; if they are not available, then you must compensate, and the function tells you which movements are possible, and which are not." [P6-C2]*
	Q8.2	*"So [by compensating] I save energy by not having to tense my biceps, for example, because I then push my arm across the table. O[...] I find it really exciting to make the left-right comparison." [P8-C3]*
	Q8.3	*"I always find the movement of the torso particularly exciting because I have the feeling that it always represents a measure of compensation, and if it's there, then it's actually almost the most important." [P9-C3]*

Table S3 Quotations of Theme 3: *Added value of ADL performance*

Subtheme	ID	Quote
Linking capacity and arm use profiles	Q9.1	*"It would be good if you could somehow say, OK, [the patient] is having therapy, so you can see in relation, [the patient] needs a lot more on the non-affected side. And when [the patient] is not in therapy or in the room on his own or something, [they] need it less. Or that's mealtime, then [the patient] needs it. So, for me […], it would also be very important to quantify that at certain times […], what [the patient] always does in relation to the other side." [P3-C1]*
	Q9.2	*"So I would really just be interested in the number of activities on the left and right side over the course of the day, in connection with the time when they happened, so that I really have an activity profile of the patient for both sides so that I can see […], perhaps also via online rehabilitation, to increase the use of more of the affected side […]. Then I want to see how actively, how much you actually use the more affected arm compared to the other, and how much you use it in absolute terms." [P1-C1]*
	Q9.3	*"[I would like to know] the activities that are most frequently used by the patient, which are restricted and from which [the patients] can benefit by improving this function, so that I have parameters in the therapy and can work on them." [P8-C3]*
	Q9.4	*"The radius that [the patient] can reach, especially as a quadriplegic with [the] hand or arm, is much more relevant for me, or is much, much more relevant for the patient in everyday practice. Where do I reach, and for what reason? [P11-C3]"*
	Q9.5	*"It is often a question of whether someone can reach shoulder height, and that is decisive as to whether the arm is subsequently used at an activity level. Just as with the hand, whether there is a certain amount of gripping strength is decisive for whether the hand is used at an activity level and ultimately also at a participation level." [P4-C2]*
	Q9.6	*"Efficient movement will carry over in everyday life because it just doesn't fatigue very much, it's efficient, it's not so slow that it's such a possible achieved goal for arm function." [P6-C2]*
Efficiency and endurance	Q10.1	*"Efficient movement will carry over into everyday life because it doesn't fatigue much; it's efficient and not so slow that it becomes a goal for arm function." [P6-C2]*
	Q10.2	*"Maybe because activity is efficient, so if the patient has to try 10 times to lift the fork, then [they] give up, so there has to be a certain efficiency of movement, otherwise [they] won't do it." [P6-C2]*
	Q10.3	*"Depending on [how the patient] can eat, it can take 5 minutes, or it can take an hour and a half, but probably, if there is an activity taking place, [the patient] must be able to at least comb [their] hair for a few minutes. If it keeps falling out of [their] hand after a few seconds, then [they] don't need [their] hand for these activities." [P6-C2]*
Contextual factors	Q11.1	*"There are a lot of personal factors, I would say. How quickly someone gets tired, how willing someone is to try again despite being tired or to give up quickly." [P4-C2]*
	Q11.2	*"There would also be factors such as cognition, motivation, and a few other factors that can also influence movement or the use of movement." [P10-C3]*
	Q11.3	*"It would be very relevant for our patients in particular to include wheelchair use. Not simply for mobility but depending on which aids the patient uses he/she has not the same […] direction of upper limb movement [that] they use." [P10-C3]*

Table S4 Quotations of Theme 4: Individual prioritization by relevance and feasibility

Subtheme	ID	Quote
Profiling patients	Q12.1	*“In our patient clientele, I would say this leans more towards highly individualized functions rather than a one-size-fits-all model”. [P8-C3]*
	Q12.2	*“So, especially in the area of movement transitions [for example] from lying down back into the wheelchair. I would say there are x variations per patient, and I think [...] individualized [assessments] just for the part of everyday life, I think most of the time [assessing] one direction of movement would not be enough.” [P11-C3]*
Individualizing what matters	Q13.1	*“The activities most frequently used by the patient are those that are restricted and can benefit their function, allowing me to set parameters in the therapy and work on them.” [P8-C3]*
	Q13.2	*“Because maybe I can see exactly when I see [...]during this free time, [the patient] was playing with the grandchildren, or reading a book, and was using the non-affected side, so only then can I somehow practice for the therapy that I can teach the non-affected side how to open a book. For example, if someone sees that it's more unilateral [use], so only [uses] the unaffected side, [...] how do I actually adapt my therapy, so in the sense of whether I should use CIMT [Constrained induced Movement Therapy] later.” [P3-C1]*
	Q13.3	*“Distinguish between capacity and performance. I mean, we can do so many assessments and evaluate activities, but if they are not used due to learned non-use, then we have to start somewhere else.” [P4-C2]*
	Q13.4	*“And even if you have two identical diagnoses, you will arrive at different measurements. I think it's the context that determines what is really important for someone. And where they might need to get some outside help or really do it themselves. It's more, it's very, very individual.“ [P10-C3]*
Balancing relevance and feasibility	Q14.1	*“I think you also have to think about the clinic [...], what can be measured in everyday clinical practice, or what is realistic here in a laboratory? [...] What is too much to ask and what is not feasible? Ideally, we would like to measure everything from the patient. To somehow generate data and be able to make statements about how the function might develop.” [P9-C3]*
	Q14.2	*“That is always included, of course, for the lower forces, making it difficult to assess how the mobility is. But if we have someone from MRC [Medical Research Council] 3 to 5, then we can say, ok, he can do the movement. They can perform a part of the movement or perform it completely over the entire range of motion.” [P9-C3]*
	Q.14.3	*“I would also imagine that when I take a kinematic measurement, it is more likely to be used in an activity than when we take muscle status or something like that, that we really just proceed in a completely standardized way, because I always find that what we measure muscle status does not mean that a patient can really use it in the activity.” [P10-C3]*

Table S5 Quotations of Theme 5: *Blending clinical eye and reference data*

Subtheme	ID	Quote
Subjective evaluation	Q15.1	*"I have a lot of [experience in] visual observation. And then the question is, do the values that I see here on paper help me to interpret them better" [P9-C3]*
	Q15.2	*"Individual movements are interesting to find out where there's a problem, but I don't really need time for that; I can see that when I do the test." [P5-C2]*
	Q15.3	*"We're just looking at how he (the patient) does execute an upper limb movement, how he finishes the action, or how he starts it, right?" [P7-C2]*
	Q15.4	*"And [the question is] can you really see that at the end, or can you really extract it from the data? Well, I also regard jerk, knowing that it can be easily measured. And then the question is whether that's really, um, whether it's not just an observation, or whether you can also see it from the data. So, I think that could also be difficult to map. It would definitely be interesting and relevant." [P11-C3]*
Objective reference values	Q16.1	*"In the case of a stroke, I find it useful to compare left with right, because I know that one side has the norm, or corresponds to the norm of mobility. With our patients (SCI), I prefer to examine one side specifically and then compare it, not with the other side of the body, but with normal values collected from a healthy control group. In order to be able to assess it, the shoulder flexion is now restricted by such and such a percentage." [P10-C3]*
	Q16.2	*"Then, you need the same function as a measurement, so that you have a standard and think: Hey, that's how far away you are from it. And that reflects the activities." [P11-C3]*
	Q16.3	*"So, what is the norm, the norm for mobility or muscles anyway? No, not for muscle activity, but for mobility, for example. I think that makes more sense, for example, when it comes to the duration of an activity. Also, the intensity, actually" [P8-C3]*
	Q16.4	*"This is actually also something that can be standardized to some extent. And perhaps carry it out with a larger group. I see the goal as always having to be set very individually, but I also have the feeling that if you decide on an activity from a research perspective, for example, then that is certainly one of the most relevant that I would want to do." [P9-C3]*
	Q16.5	*"But I still find it exciting when you and SCI are both affected. You may have had hand dominance before the accident and so on. And comparing all of these aspects, how is the use of the arm in everyday life, I find this aspect exciting. Logically, if you also compare it with the healthy cohort, then you can actually say you are restricted or not." [P9-C3]*
	Q16.6	*"But what is commonly used in everyday life, in other words, what is the comparative image? Imagine: if […] I have a kinematic measurement for the range of motion of the arm. I would need to know whether this is the norm or whether it deviates. Being able to interpret that again is then somehow also difficult." [P9-C3]*

Table S9 continued

Subtheme	ID	Quote
Integrating complex interactions	Q17.1	*"I think that the coordination between the shoulder joint, the elbow, and the interaction, uhm, of all, yes, 3 joints is decisive for which function I can ultimately perform. So I think that if you have a poor shoulder connection" [P10-C3]*
	Q17.2	*"From coordination to inter-joint coordination or perhaps multi-joint synergies in stroke. I can imagine that... so I can see that, too, but just now. Yes, I'm supposed to derive straight forwardness as a very complex, how do you really derive it from a measurement?" [P9-C3]*
	Q17.3	*"If you have a gripping movement now and imagine that you can do this with, if I see how elegantly I am gripping the cup directly, but it can be, for example […] that I wobble a lot, that can be due to a force deficit, but it can also be a lack of control that I don't know exactly yet." [P2-C1]*
	Q17.4	*"Yes, although it can be the case that you try, for example, to use all the muscles individually, i.e., intra-limb, but you can also use them within a movement, so even if you control the muscles individually in the correct sequence, you may have a poor estimate of the force that you need to apply. Stroke patients, for example, always show overshooting in many tasks." [P2-C1]*
	Q17.5	*"Ultimately, if you don't know where the hand is in space, you can't grasp the glass and being dependent […] on eye vision when grasping things – this is certainly an issue in SCI patients - and then also not being able to feel it and then not grasping it properly. So, we definitely have this dependency in SCI patients in this area. These are influencing factors, and the structures will influence the others." [P11-C3]*
	Q17.6	*"I think for everyday activities, the question for me is always what the ratio of strength to mobility is. And yes, we already have that in the MRC, yes. But I mean, you measure the range of motion where the patient is in this movement environment, which means it's never the global one. If it's only reduced, I measure it in that too and make a note of it." [P11-C3]*
	Q17.7	*"And that would be the big difference to a kinematic movement quality measurement, that you can really look at the activity that is meaningful for the patient, okay, what is available to him, and why perhaps? Why are there perhaps deviations from the standard test and then in the activity?" [P10-C3]*
