# Supplementary material for: From Metrics to Meaning in Neurological Rehabilitation: Clinicians’ Perspectives on Digital Metrics of Upper Limb Functioning—A Focus Group Study
Source: JMIR Rehabil Assist Technol. 2026 Jun 24;13:e87339. doi: 10.2196/87339 (PMC13293569; doi:10.2196/87339)
Supplement: Checklist 1 [file rehab-v13-e87339-s004.pdf]

Standards for Reporting Qualitative Research (SRQR)

No.	Topic	Item	Location in Manuscript
S1	Title	Concise description of the nature and topic of the study identifying the study as qualitative or indicating the approach (e.g., ethnography, grounded theory) or data collection methods (e.g., interview, focus group) is recommended	Title page
S2	Abstract	Summary of key elements of the study using the abstract format of the intended publication; typically includes background, purpose, methods, results, and conclusions	Abstract
S3	Problem formulation	Description and significance of the problem/phenomenon studied; review of relevant theory and empirical work; problem statement	Introduction
S4	Purpose or research question	Purpose of the study and specific objectives or questions	Introduction
S5	Qualitative approach and research paradigm	Qualitative approach (e.g., ethnography, grounded theory, case study, phenomenology, narrative research) and guiding theory if appropriate; identifying the research paradigm (e.g., postpositivist, constructivist/interpretivist) is also recommended; rationale	Methods- study design
S6	Researcher characteristics and reflexivity	Researchers' characteristics that may influence the research, including personal attributes, qualifications/experience, relationship with participants, assumptions, and/or presuppositions; potential or actual interaction with participants during study; and how these were managed	Methods-procedures
S7	Context	Setting/site and salient contextual factors; rationale	Methods-procedures
S8	Sampling strategy	How and why research participants, documents, or events were selected; criteria for deciding when no further sampling was necessary (e.g., sampling saturation); rationale	Methods-participation and recruitment
S9	Ethical issues pertaining to human subjects	Documentation of approval by an appropriate ethics review board and participant consent, or explanation for lack thereof; other confidentiality and data security issues	Methods-study design
S10	Data collection methods	Types of data collected; details of data collection procedures including (as appropriate) start and stop dates of data	Methods - procedure

		collection and analysis, iterative process, triangulation of sources/methods, and modification of procedures in response to evolving study findings; rationale	
S11	Data collection instruments and technologies	Description of instruments (e.g., interview guides, questionnaires) and devices (e.g., audio recorders) used for data collection; if/how the instrument(s) changed over the course of the study	Methods - procedure
S12	Units of study	Number and relevant characteristics of participants, documents, or events included in the study; level of participation (could be reported in results)	Methods -Table1
S13	Data processing	Methods for processing data prior to and during analysis, including transcription, data entry, data management and security, verification of data integrity, data coding, and anonymization/deidentification of excerpts	Methods- analysis
S14	Data analysis	Process by which inferences, themes, etc., were identified and developed, including the researchers involved in data analysis; usually references a specific paradigm or approach; rationale	Methods- analysis
S15	Techniques to enhance trustworthiness	Techniques to enhance trustworthiness and credibility of data analysis (e.g., member checking, audit trail, triangulation); rationale	Participant checking, Methods
S16	Synthesis and interpretation	Main findings (e.g., interpretations, inferences, and themes); might include development of a theory or model, or integration with prior research or theory	Results
S17	Links to empirical data	Evidence (e.g., quotes, field notes, text excerpts, photographs) to substantiate analytic findings	Results
S18	Integration with prior work, implications, transferability, and contribution(s) to the field	Short summary of main findings; explanation of how findings and conclusions connect to, support, elaborate on, or challenge conclusions of earlier scholarship; discussion of scope of application/generalizability; identification of unique contribution(s) to scholarship in a discipline or field	Discussion
S19	Limitations	Trustworthiness and limitations of findings	Limitations
S20	Conflicts of interest	Potential sources of influence or perceived influence on study conduct and conclusions; how these were managed	Acknowledgements
S21	Funding	Sources of funding and other support; role of funders in data collection, interpretation, and reporting	Acknowledgements
